# Sociomicrobiome of wood decay in a tropical rain forest: unraveling complexity

**DOI:** 10.1186/2193-1801-2-435

**Published:** 2013-09-04

**Authors:** Tasha M Santiago-Rodriguez, Gary A Toranzos, Paul Bayman, Steven E Massey, Raul J Cano

**Affiliations:** Biology Department, University of Puerto Rico, San Juan, PR 00932 USA; California Polytechnic State University, San Luis Obispo, CA 93407 USA; Center for Applications in Biotechnology, San Luis Obispo, CA 93407 USA

**Keywords:** Phenazines, Quorum-sensing, Quorum-quenching, Resistance, Secondary metabolites, Toxin-antitoxin systems

## Abstract

Given that microbial interactions in nature are very complex, we propose that quorum-sensing, as well as quorum-quenching, phenazine and secondary metabolite production, resistance and toxin-antitoxin systems within a microbial community should all comprise the battery of processes involving the study of what we would define as the “sociomicrobiome”. In the present study the genes/molecules, subsystems and taxonomic breakup of the mentioned processes were identified in decaying tropical wood from the El Yunque rainforest in Puerto Rico, and soil using a shotgun metagenomic approach. The rapid decomposition of wood and litter in tropical regions suggests that processes in these settings are governed by unexplored microbes with the potential of being further studied and exploited for various purposes. Both ecosystems were characterized by the presence of specific genes/molecules, subsystems and microbes associated with the mentioned processes, although the average abundances for specific processes differed. Of the sociomicrobiomes studied, that from El Yunque was found to be the most complex. The approach considered in the present study could also be applied to study the sociomicrobiome of other ecosystems.

## Introduction

There is an increasing interest towards microbial communication in natural settings (Furhman, 2009). Despite being a highly complex process, microbial communication has been mainly associated with quorum-sensing (QS) (Bassler, 1999). Microbial communication is mediated by acylated homoserine lactones (AHLs) and autoinducer-2 (AI-2) in bacteria, and farnesol in fungi (Decanis et al., 2011;Winker et al., 2003). Aromatic compounds and compounds that have antibiotic activity may also be involved in microbial communication in natural settings, although the latter remains a matter of further research (Romero et al., 2011). Microbial communication can be disrupted by acylases, lactonases or nucleosidases, a process known as quorum-quenching (QQ) (Sio et al., 2006). Other molecules, such as the phenazines, can act in promoting or disrupting microbial communication (Pierson and Pierson, 2010). More interesting perhaps is the effect that microbial communication may have on gene expression. This in turn may alter the abundance and diversity of microbial communities in natural settings. An example of such genes is the toxin-antitoxin (TA) systems, essential for various processes in bacteria (Engelberg-Kulka et al. 2005), (2006).

Most studies so far have focused on specific molecules involved in microbial communication and thus often ignore the overall complexity of this process. QS, QQ, secondary metabolite and antibiotic production, and TA systems are part of the complex microbial interactions in nature. We believe that a battery of the processes described in the present study can be attributed to what we would call the “sociomicrobiome”. Sociomicrobiome will be defined as the microbes (bacteria and fungi) and the genes/molecules involved in promoting or disrupting communication in environmental processes. As we define it, studies of the sociomicrobiome of an ecosystem may provide better insights into the ecology and “social behavior” of microorganisms, both within and between species. Similar terms were originally coined to define the association of QS with biofilm formation (as in the case of “sociomicrobiology”), and the abundance of microbes, their genes and interactions in an ecosystem (as in the case of “microbiome”) (Parsek and Greenberg, 2005;Gill et al., 2006). Studies regarding the microbiome consider mostly the abundance and diversity of bacteria (Gill et al., 2006). These studies often ignore the role of other microbes (e.g. fungi) and the genes/molecules involved in their interactions with the surrounding environment and as possible regulators of specific population sizes within a community.

Decaying wood and soils could be a reference for studies regarding the sociomicrobiome, but it should be noted that any ecosystem may possess its unique sociomicrobiome. Processes taking place in these settings are characterized by unique and complex microbial communities (DeAngelis et al., 2011). Tropical wood and humid tropical forest soils have fast decomposition rates, suggesting that wood decomposition, as well as processes in soils present in tropical regions are governed by microorganisms with novel and specific pathways (DeAngelis et al., 2011). However, little is known in terms of the source of these microorganisms, which in many cases degrade complex polymers in a specific order; we hypothesize that most, if not all of these microorganisms are present at all times in the ecosystem being studied. It would seem that the cell/species densities change, as conditions/substrate types change. Thus, all of these microorganisms would have to respond to a “social environment” modulated by the cells, conditions and substrates present. In the present study, we determined the abundance of genes and molecules involved in microbial communication (AHL synthases, LuxR, AI-2, TraR and phenazines), inhibition of such (AHL acylases, AHL lactonases, AI-2 nucleosidases and *cdr1*), antibiotic-resistance (as a possible indicator of the production of antibiotics in nature), secondary metabolite production and TA systems in decaying wood and tropical soils by using a comparative metagenomics approach as a first step in the elucidation of a sociomicrobiome present in ecosystems where complex polymers are being degraded.

## Materials and methods

### Sample description and preparation

The sample (EY log) was collected at the El Verde Field Station of the UPR, located in the El Yunque National Forest in northeastern Puerto Rico (18°20' N, 65°49' W). It is a mature secondary forest classified as Subtropical Wet in the Holdridge Life Zone classification, about 550 masl and with 3500 mm annual rainfall (Thompson et al. 2002), and is the only tropical rainforest in the US National Forest system. It is a highly diverse forest dominated by tabonuco (*Dacryodes excelsa*, Burseraceae). Tabonuco is an evergreen tree to 40 m notable for production of terpinoid-rich resin. It is likely that the log we sampled is *D. excelsa*, based on wood color and texture and location, but the log was too degraded to be identified.

Genomic DNA was extracted using the PowerSoil® DNA Isolation Kit (Mo Bio Laboratories, Carlsbad, CA), following the manufacturer’s instructions. Three replicate DNA samples were pooled in order to obtain a homogeneous representation of DNA. DNA concentration and quality were estimated spectrophotometrically and by agarose gel electrophoresis in a 0.8% agarose gel, respectively.

### Metagenome sequencing and analysis

Purified genomic DNA from the EY log was prepared for shotgun metagenomics library construction as indicated by the DOE Joint Genome Institute standard operating procedure for shotgun sequencing using the Roche 454 GS FLX Titanium technology (Branford, CT). Raw sequence data was prepared for uploading into the MG-RAST server by removing: replicates; first four nucleotides of each sequence; sequences with average q value < 15; sequences with more than 10 Ns; sequences with a tail of ≥ 10 Ns; and sequences with % N > 4. Following this preliminary quality filtering the raw FASTA file was uploaded in the MG-RAST server for further filtering, analysis and annotation. The filtering parameters used are summarized in Table 1.

**Table 1:** **Filtering parameters used for El Yunque log metagenome**

Parameter	Description	
Length filtering	Standard deviation multiplicator for length cutoff	2.0
Ambiguous base filtering	Maximum number of allowed ambiguous base pairs	5
Dynamic trimming	Lowest phred score counted as high quality base	15
	Trim number of bases in low quality sequences	5
Dereplication	Remove artificial replicate sequences	N/A
Screening	Remove host-specific sequences	H. sapiens NCBI v36

Searches for each gene/molecule and functions were performed in the decaying log metagenome and a metagenomic dataset derived from soil obtained from Luquillo, in the foothills of the El Yunque forest (MG-RAST ID 4446153.3) using MG-RAST. Similarity searches at MG-RAST allow retrieving similarities to databases such as NCBI, PATRIC, KEGG, SEED and RefSeq. Results were acquired by the “Best Hit” and “Search Function” options. QS and QQ gene function in both ecosystems were compared using Multidimensional scaling (MDS) analyses, constructed using a resemblance matrix of Bray-Curtis similarities (Primer E software v. 6) (Clarke and Gorley, 2006). All data considered in the present study are freely available in MG-RAST (e.g. Waseca soil, Waseca County, Minnesota, United States) (Table 2).

**Table 2:** **Comparative taxonomic and functional diversity metrics for selected metagenomes**

Biome	Sample	MG-RAST ID	S^1^		d^2^		Fisher’s α		H' (loge)^3^	
			Taxon	Metab^4^	Taxon	Metab	Taxon	Metab	Taxon	Metab
**Wood**	El Yunque log	4491607.3	790	22	84.52	4.50	193.30	8.44	5.84	2.79
**Soil**	Agricultural soil	4441091.3	409	17	46.94	3.95	99.60	8.15	5.50	2.60
	Temperate soil	4453261.3	640	22	66.24	4.03	134.70	6.52	5.67	2.71
	Tropical soil	4446153.3	598	22	63.89	4.30	134.20	7.52	5.65	2.73
	Compost	4449125.3	461	19	48.01	3.32	90.72	4.93	5.19	2.46
**Aquatic**	Coral reef atoll	4441604.3	559	11	63.32	2.42	144.90	3.88	5.69	2.15
	Antarctic Lake	4443685.3	371	4	50.34	1.08	154.10	1.61	5.61	1.36
**Microbial Mats**	Stromatolite	4449590.3	521	10	64.59	2.87	178.30	6.75	5.78	2.20
	Biofilm	4460448.3	532	15	57.71	3.32	120.30	5.94	5.38	2.14
**Animal**	Cow rumen	4441681.3	338	7	40.80	1.64	89.10	2.49	5.10	1.70
	Combined rumen	4491686.3	386	9	42.23	1.99	81.73	3.02	5.20	1.79
	Termite gut	4442701.3	425	7	51.25	1.79	121.20	2.97	5.60	1.52
	Canine gut	4444703.3	418	8	47.11	1.90	97.60	3.02	5.07	1.73
	VW Pig gut	4461380.3	387	4	48.45	1.09	120.30	1.75	5.33	1.30

^1^S = total species.

^2^Margalef’s species richness where d = (S-1)/log_e_N for S = total number of species and N = total individuals.

^3^Shannon Diversity Index where H’ = - Σ Pi log_e_(Pi).

^4^Diversity metrics based on abundance of secondary metabolism genes in metagenome.

## Results

### Uniqueness of decaying wood

To put the diversity of the EY metagenome in perspective, it was compared to shotgun metagenomes from soil, aquatic systems, mats, and gut microbiota (Table 2). Although these metagenomes differ in substrate, sampling depth, study design and methodology, some trends are apparent. The EY metagenome was highest in number of species predicted, Margalef’s species richness index, and Fisher’s ɑ and Shannon’s diversity indices. Both Fisher's ɑ and Shannon’s diversity indices take account of richness and evenness, but Shannon’s is more sensitive to richness. This is especially relevant for metagenomes, whose richness is generally higher than traditional biodiversity studies because of sampling depth. As Table 2 shows, both predicted richness and evenness in a rotting log are comparable to or higher than better-known, complex microbial systems. A point to note is that taxonomic diversity may be influenced by bias in the microbial genomes present in the database, given that for shotgun metagenomes taxonomic affiliation is determined by similarity to sequences present in the non-redundant database, given differences in the representation of different microbial groups in the different samples. This concern will be reduced as a more even sequencing coverage of microbial taxa is accomplished. An additional strategy to characterize complexity is to assess the diversity of the enzymes involved in secondary metabolism. Here, homologs present in each sample can be determined with a degree of certainty and their function inferred from sequence similarity. Biases in terms of genes present in the database are likely to affect all taxa in the samples equally. Given the taxonomic richness and evenness, microbial crosstalk is not surprising. When just the environmental metagenomes were considered, there was a significant, negative correlation between Shannon’s index and latitude (R2= 0.54, F=8.30, P=0.02). Intriguingly, this mirrors the negative correlation observed between plant and animal diversity and latitude. However, further work will be necessary to verify this observation, as it appears to contradict previous results on microbial diversity (Fierer and Jackson 2005).

### Quorum-sensing and quorum-quenching taxonomic breakup

**Figure 1:**
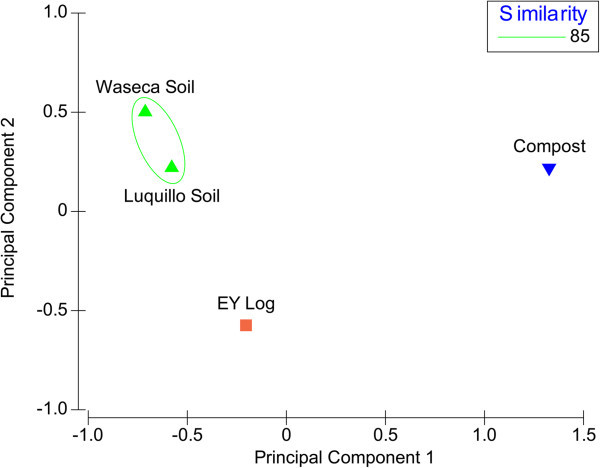
**Principal component analysis of quorum-sensing and quorum-quenching subsystems in decaying log, soils and compost.**

QS and QQ genes function subsystems were compared to those of the Luquillo and Waseca soils and composites (Figure 1). The subsystems from the Luquillo and Waseca soils showed a similarity of 85% and the composite and EY log grouped separately. Taxonomic breakup (by class and species) of the QS and QQ genes in the EY log and Luquillo soil are presented in Table 3. The Alphaproteobacteria, in both the decaying log and tropical soil, accounted for sequences encoding for AHL synthases and LuxR. Sequences in the decaying log were similar to that present in *Phenylobacterium zucineum*, while *luxR* was similar to that present in *Sinorhizobium fredii* (61). In the tropical soil, species related to *Rhodobacter sphaeroides* and *Sulfitobacter* spp. accounted for the AHL synthase- and LuxR-encoding genes. The Alphaproteobacteria, Gammaproteobacteria, Actinobacteria and Cytophagia accounted for the AHL acylases and lactonases and AI-2 nucleosidases sequences in the decaying log. The Alphaproteobacteria, Betaproteobacteria, Gammaproteobacteria and Actinobacteria harbored genes encoding for QQ molecules in the tropical soil. In terms of *cdr1*, detection was restricted to the decaying log.

**Table 3:** **Taxonomic breakdown of quorum-sensing and quorum-quenching genes in decaying log and tropical soil metagenomes**

Molecule/Receptor	Class	Species	Abundance	e-value	Average identity (%)
**Quorum-sensing and quorum-quenching decaying wood**					
**AHL synthase**	Alphaproteobacteria	Phenylobacterium zucineum	1816	-22.45	70.78
**LuxR**	Alphaproteobacteria	Sinorhizobium fredii	707	-22.73	70.03
**AHL acylase**	Alphaproteobacteria	Citromicrobium bathyomarinum	243	-18.77	68.96
	Gammaproteobacteria	Pseudomonas aeruginosa	279	-21.83	68.39
**AHL lactonase**	Actinobacteria	Rhodococcus erythropolis	169	-16.22	69.00
**AI-2 nucleosidase**	Alphaproteobacteria	Asticcacaulis excentricus	1172	-21.00	71.41
	Cytophagia	Dyadobacter fermentans	736	-16.32	68.38
***cdr1***	Eurotiomycetes	Arthroderma gypseum	111	-14.55	71.01
**Quorum-sensing and quorum-quenching tropical soil**					
**AHL synthase**	Alphaproteobacteria	Rhodobacter sphaeroides	600	-20.00	67.60
	Alphaproteobacteria	Sulfitobacter sp. NAS-14.1	201	-16.97	67.99
**LuxR**	Alphaproteobacteria	Rhodobacter sphaeroides	600	-20.00	67.60
	Alphaproteobacteria	Sulfitobacter sp. NAS-14.1	201	-16.97	67.99
**AHL acylase**	Gammaproteobacteria	Pseudomonas fluorescens	589	-18.90	66.67
	Gammaproteobacteria	Pseudomonas putida	288	-22.18	67.29
**AHL lactonase**	Actinobacteria	Rhodococcus erythropolis	305	-19.24	66.91
		Rhodococcus opacus	512	-18.72	67.73
**AI-2 nucleosidase**	Betaproteobacteria	Burkholderia graminis	683	-21.89	68.39
	Betaproteobacteria	Burkholderia sp. CCGE1001	320	-20.97	67.33
	Betaproteobacteria	Variovorax paradoxus	1184	-20.16	68.73
***cdr1***	ND	ND	ND	ND	ND

ND=not detected.

**Figure 2:**
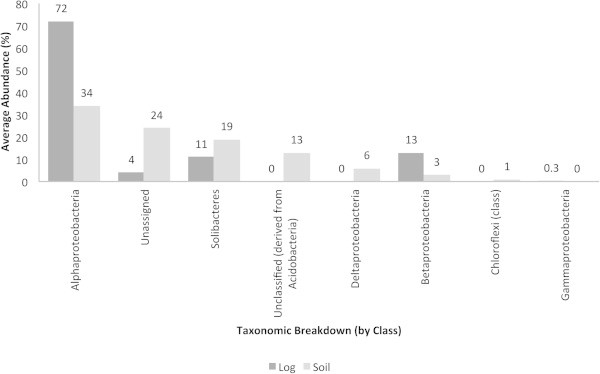
**Average abundances (%) of traR in decaying log and tropical soils.** Taxonomic breakup was performed by class.

**Figure 3:**
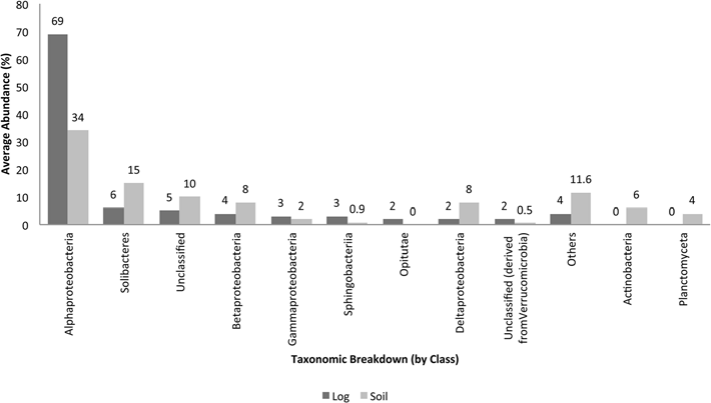
**Average abundances (%) of AI-2 synthases in decaying log and tropical soils.** Taxonomic breakup was performed by class.

In contrast to the mentioned QS and QQ molecules, *traR* (Figure 2) and AI-2 synthases (Figure 3) were detected in more bacterial groups. In the decaying log and soil, the Alphaproteobacteria accounted for the highest abundance of *traR* (70 and 35%, respectively). In soil, particularly, unclassified bacteria (derived from the Acidobacteria), the Deltaproteobacteria and Chloroflexi accounted for the highest abundances of *traR*. The Alphaproteobacteria were the most abundant group harboring genes responsible for the synthesis of AI-2 in the decaying log (70%) and soil (35%), followed by the Solibacteres. The decaying log was characterized by the Opitutae and unclassified bacteria (derived from the Verrucomicrobia) and both groups did not show to harbor AI-2 genes in soil. The Actinobacteria and Planctomyceta accounted for AI-2 synthase genes in soil, particularly.

### Taxonomic breakup of phenazines

**Figure 4:**
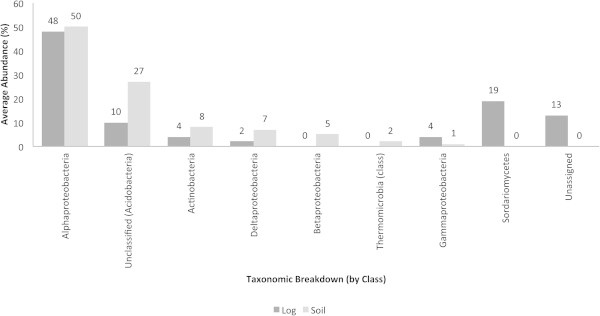
**Phenazines average abundances (%) in decaying log and tropical soils.** Taxonomic breakup was performed by class.

The taxonomic breakup (by class) of phenazines in the decaying log and soil are presented in Figure 4. The Alphaproteobacteria accounted for the highest abundance of phenazines in the decaying log and soil (48 to 50%). In both ecosystems, unclassified bacteria (derived from the Acidobacteria), Actinobacteria and Deltaproteobacteria were responsible for the presence of genes encoding for phenazines as well. In the decaying log, these bacterial groups accounted for lower abundances of phenazine genes when compared to soil. The Betaproteobacteria and Thermomicrobia (< 5%) accounted for the presence of phenazine genes in soil. Interestingly, fungi from the Sordariomycetes, a group involved in wood decomposition, harbored phenazine genes in the decaying log and this was not noted for the soil sample.

### Metabolism of aromatic compounds subsystems and taxonomic breakup

**Figure 5:**
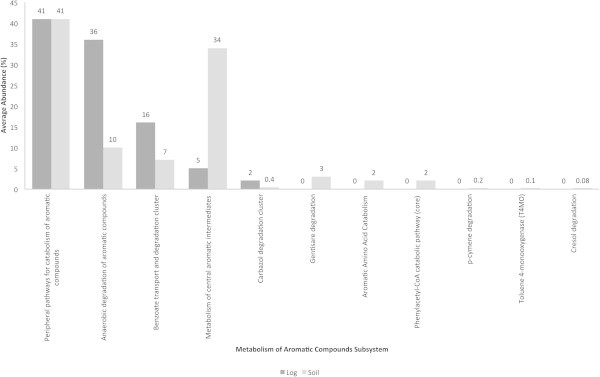
**Metabolism of aromatic compounds in decaying log and tropical soils.** Average abundances (%) of the subsystems are shown.

Metabolism of aromatic compounds subsystems are shown in Figure 5. Genes responsible for peripheral pathways for catabolism of aromatic compounds in the decaying log and soil were present in an average abundance of 40%. The average abundance of sequences accounting for the anaerobic degradation of aromatic compounds in the decaying log and soil were 35 and 10%, respectively. Benzoate transport and degradation genes were detected in average abundances of 15 and 7% in the decaying log and soil, respectively. Interestingly, genes accounting for the metabolism of central aromatic intermediates were present in higher abundances in soil (35%), compared to the decaying log (5%).

**Figure 6:**
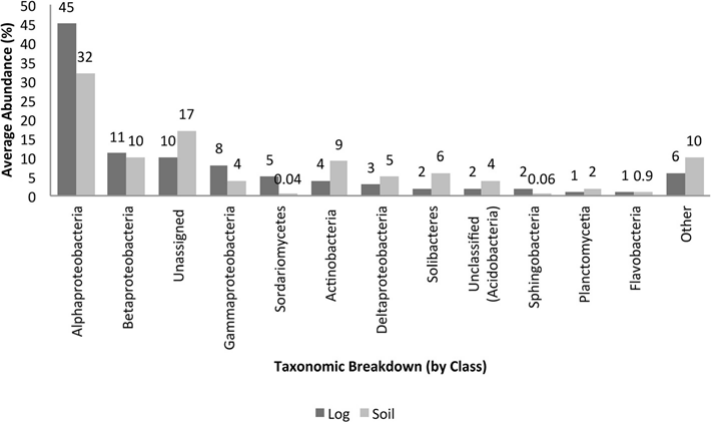
**Average abundances (%) of microorganisms involved in the metabolism of aromatic compounds.** Taxonomic breakup was performed by class.

The taxonomic breakup (by class) of genes involved in the metabolism of aromatic compounds is shown in Figure 6. In these subsystems, the Alphaproteobacteria accounted for the highest abundances in the decaying log (45%) and soil (32%), followed by the Betaproteobacteria (approximately 10%) and unassigned bacteria (10 to 15%). Similarly to phenazines in the decaying log, the Sordariomycetes harboring genes involved in the metabolisms of aromatic compounds were only present in the decaying log.

### Resistance subsystems and taxonomic breakup

**Figure 7:**
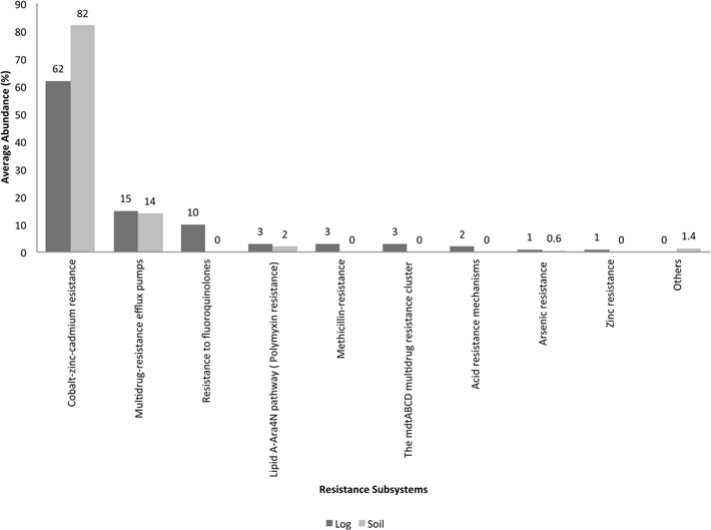
**Resistance subsystems in decaying log and tropical soils.** Average abundances (%) of the subsystems are shown.

Various resistance subsystems were present in both the decaying log and soil (Figure 7). Subsystems of cobalt-zinc-cadmium resistance accounted for the highest abundances in both the decaying log (approximately 20%) and soil (40%). Sequences accounting for the cation efflux system protein CusA were present in the decaying log and soil (approximately 20 and 17%). Polymyxin resistance protein ArnT was present in both ecosystems as well (<1%). Interestingly, acriflavin resistance was only present in the decaying log (approximately 10%). Other resistance subsystems were present in both ecosystems in abundances of <1%, but overall, these accounted for approximately 35 and 10% of the resistance gene sequences in the decaying log and soil, respectively.

**Figure 8:**
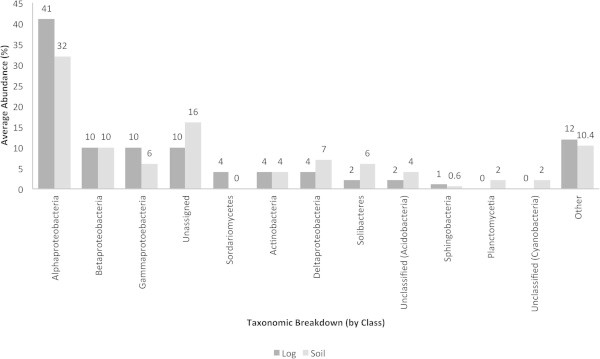
**Average abundances (%) of microorganisms exhibiting resistance mechanisms.** Taxonomic breakup was performed by class.

The taxonomic breakup (by class) of microbes responsible for the resistance subsystems are shown in Figure 8. The Alphaproteobacteria (41 and 32%), Betaproteobacteria (10%), Gammaproteobacteria (10 and 6%) and unassigned (10 and 16%) bacteria accounted for the highest abundances of resistance subsystems in the decaying log and the tropical soil. Interestingly, the Sordariomycetes (4%) were restricted to the decaying log, while the Planctomycetia and unclassified bacteria derived from the Cyanobacteria were restricted to soil (2%).

### TA subsystems

**Figure 9:**
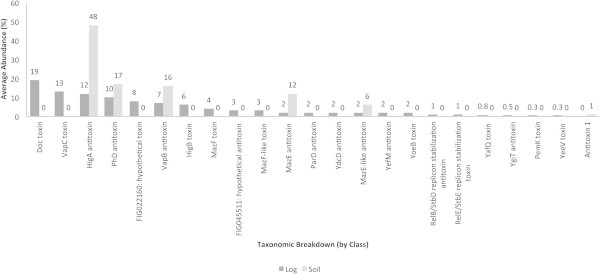
**Toxin-antitoxin (TA) gene subsystems in decaying log and tropical soils.** Average abundances (%) of the subsystems are shown.

The decaying log exhibited the presence of more TA system subsystems compared to the tropical soil (Figure 9). The toxin part of the PhD/Doc and VapC/VapB systems represented the highest abundances of TA toxins in the decaying log and soil (20 and 15%, respectively). Although the Doc and VapC toxins were not detected in the tropical soil, the antitoxin part, PhD and VapB, were present in this ecosystem (approximately 18%), as well as in the decomposing log (approximately 8 to 10%, respectively). Interestingly, the HigA antitoxin accounted for the highest abundance of a TA system subsystem in soil (50%) (10% in the decaying log), while the toxin part, HigB, was not detected in soil. A similar outcome was noted with the MazEF and MazEF-like TA systems, characteristic of *Escherichia coli*, in which the antitoxin part was detected in soil, while the toxin element was absent.

**Figure 10:**
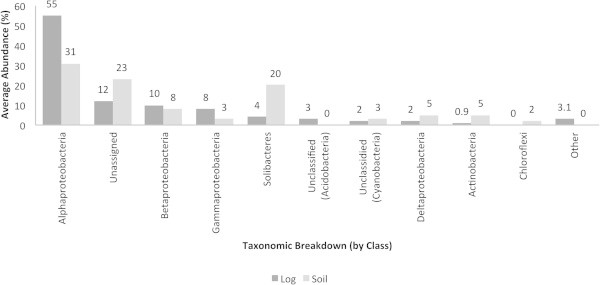
**Average abundances (%) of microorganisms harboring TA systems.** Taxonomic breakup was performed by class.

As with other genes/molecules in the present study, the Alphaproteobacteria accounted for the highest abundances of TA systems in the decaying log and soil (Figure 10). Unassigned, Betaproteobacteria and the Gammaproteobacteria are among the groups harboring TA systems in both ecosystems. Unclassified bacteria (Acidobacteria) and Chloroflexi accounted for TA systems specifically in the decaying log and soil, respectively.

## Discussion

The present study determined the abundance of genes/molecules, subsystems and taxa of microbes involved in wood decomposition and soil in an attempt to provide a wider view of the possible interactions. The distinctiveness of the subsystems in the decaying log (when compared to soil), clearly supports the uniqueness of the microbial communities in this setting. Moreover, the separate clustering of the EY log and composite sample supports the uniqueness of the decaying wood as well. Composite was chosen as a reference sample microbial processes in this type of sample could be very similar to those taking place in decaying wood (Miyatake and Iwabuchi, 2006). Although this was not the case, it is possible that the microbial communities at another step of composite decomposition may be similar to the microbial processes in decaying wood. Notably, calculated diversity indexes suggest that microbial diversity is generally higher in the tropics. However, this point is still unresolved; latitude may place an upper limit on microbial diversity but is not believed to be the primary determinant. QS subsystems of tropical soils exhibited a similarity of 80% with the Waseca soil. This suggests that certain QS subsystems may be conserved while individual species may differ, and is analogous to the conservation of homologous protein structures, while the respective amino acid sequences may vary considerably.

Wood is structurally complex, and its decomposition involves a succession of different groups of organisms. Degradation of components of wood by one organism provides substrates for other organisms (for example, cleavage of lignocellulose by white rot fungi liberates sugars and makes other components accessible), so it can be viewed as a collaborative process. Furthermore, there is spatial delimitation of territory by competing organisms, often intraspecific, and is easily observed as melanized lines of interaction in decaying wood. Parasitism of one guild of wood-decay organisms by others is also common. Given this combination of succession, collaboration, competition and parasitism, it is not surprising that we found evidence of extensive microbial crosstalk in decaying wood.

These types of ecological interaction will depend on the net costs and benefits from the association and will change continuously. For instance, certain intestinal microbes produce metabolites for the benefit of other microbes in mutualistic interaction. It has been suggested that microbes can be “forced” into such interactions by specific mechanisms of QS. This scenario can certainly be applied to decaying wood and soils. Secondary metabolites (as those involved in QS) promote the expression of specific genes involved in processes that occur in wood decomposition and in soil. This genetic expression, which results in specific types of coordinated behaviors, may promote higher abundances of certain microbes. The reason is that the synthesis of molecules that could be beneficial for the proliferation and persistence of specific microbial populations may also be harmful for others (Zhu et al. 1998). Similar outcomes have been reported for activated sludge, in which AHLs mediate the composition and function of the microbial communities. Moreover, microorganisms may also exhibit signal-quenching mechanisms, resistance to antibiotics, toxins and other secondary metabolites, resulting in even more complex interactions. All these processes may have developed in particular microbes that act in specific pathways of wood decomposition and in complex processes in soils.

### Mechanisms of microbial communication

The present study is comparable with previous reports of the presence of AHL synthases and LuxR in the Alphaproteobacteria (Koch et al., 2005;Lindum et al., 1998). Even though the bacterial classes were identical in the decaying log and soil, the bacterial species were different. Species related to *Phenylobacterium zucineum* and *Sinorhizobium fredii* accounted for AHL synthase genes and *luxR* in the decaying log, respectively. *P. zucineum* is an intracellular facultative bacterium recently isolated from the human leukemia cell line K562 (Luo et al., 2008). A similar outcome was noted with *S. fredii*, which has been characterized as a symbiont of legumes (Krishnan et al., 2003). The presence of these microbes in the log is surprising, but could suggest a possible new role for these bacteria in wood decomposition. The roles of species related to *R. sphaeroides* or *Sulfitobacter* spp. in the tropical soil remain to be addressed as well, but these bacteria possess diverse metabolic capabilities and include photosynthesis and nitrogen fixation (as in the case of *R. sphaeroides*) and chemoorganotrophy (as with *Sulfitobacter* spp.) (Long et al., 2011;Poole et al., 1989). The particular role of species related to *S. fredii* in soil represents a matter of further research since these bacteria have been particularly characterized in marine environments. Notably, bacteria harboring AHL synthase genes did not harbor LuxR in the decaying log, and this was not the case for bacteria in the tropical soil. In soil, the same bacterial species accounted for the presence of both AHL synthase genes and *luxR*. These results suggest that the AHL synthase genes in the decaying log may be *luxI* homologues. In terms of *traR* (a homologue of *luxR*), this gene has been mainly identified in *Agrobacterium tumefaciens*, which belong to the Alphaproteobacteria. Consistent with previous studies, the highest *traR*-encoding bacterial abundances in both ecosystems corresponded to the Alphaproteobacteria. Yet, its presence in several bacterial groups was unexpected, suggesting that these sequences may be homologues of *traR* (Zhu et al., 2011;Swiderska et al., 2001). Bacterial groups harboring genes responsible for the synthesis of AI-2 were more diverse, supporting the universality of this molecule.

### Inhibition of microbial communication

More interesting is the greater diversity of bacteria harboring QQ genes. The Alphaproteobacteria (closest match to *Citromicrobium bathyomarinum*), as reservoirs of AHL acylase genes, were restricted to the decaying log, suggesting a specific role for these microbes in wood decomposition. Similarly, the Gammaproteobacteria accounted for the presence of AHL acylase genes in the decaying log (closest match to *Pseudomonas aeruginosa*) and soil (species related to *Pseudomonas fluorescens* and *Pseudomonas putida*), and similar outcomes were noted with the AHL lactonase genes, which were restricted to the Actinobacteria (species related to *Rhodococcus* spp.) in both ecosystems. In terms of the AI-2 nucleosidases, the decaying log and soil were characterized by the Alphaproteobacteria and Cytophagia, and the Betaproteobacteria, respectively. This indicates that a greater diversity of bacteria (although different groups) may be involved in quenching the signal produced by bacteria harboring genes responsible for the synthesis of AI-2.

Inhibition of AHL and AI-2 molecules may not represent the only QQ pathways in the ecosystems tested. Results showed that the metabolism of aromatic compounds is well represented in the decaying log and soil. Some aromatic compounds may serve as signaling molecules in microbial communication, as in the case of autoinducer-3, involved in interkingdom signaling; hence, degradation of such signals is feasible (Zhu et al., 1998). Notably, genes involved in the anaerobic degradation of aromatic compounds were more abundant in the decaying log compared to soil, and this is supported by the water-logged nature of the sample.

Little is known about QS in fungi, and certainly less is known about possible QQ signaling pathways. Farnesol has been associated with the overexpression of specific genes, such as *cdr1*, in fungi of clinical importance (e.g. *C. albicans*) (Westwater et al., 2005). *cdr1* encodes for efflux pumps and its over-expression is considered to be a drug resistance mechanism in *C. albicans* (Decanis et al. 2011). The presence of *cdr1* in species related to *Arthroderma gypseum* in the decaying log was surprising since this gene has been mainly associated with *C. albicans*. Cdr1 is a homologue of Mrp1, specific for mammalian cells; therefore, it remains feasible that *cdr1* related to *A. gypseum* represents a homologue of *cdr1* present in *C. albicans*.

### Phenazines as mediators of microbial communication

Phenazines are produced by many bacteria that are associated with a host, but less is known about fungi as phenazine-producers (Pierson and Pierson, 2010). In the present study, fungi from the Sordariomycetes accounted for the presence of phenazines in the decaying log and not in the soil. This suggests that the production of phenazines by fungi may be important for wood decomposition and opens the opportunity to characterize their role as phenazine-producers in natural settings. Phenazines were also present in the tropical soil tested and this is consistency with previous reports (Pierson and Pierson, 2010). In humid forests, respiration by microbes and plant roots may limit the availability of oxygen as an electron acceptor. This, in turn, is consistent with the water logged nature of the log from which the dataset was generated.

### Resistance mechanisms

Resistance could be the result of the complex microbial interactions in nature. For instance, cobalt-zinc-cadmium resistance was noted in both the decaying log and soil, with greater representation in the soil. This may reflect the higher concentrations of these divalent cations in soil. Results show that ion-resistance in the decaying log and soil is present in various microbial communities, which are not limited to bacteria as fungi have also exhibited resistance to ions (Nies, 1992). Similarly, the presence of genes encoding for multidrug-resistance in pristine environments (as those considered in the present study), supports that antibiotic production is not restricted to clinical settings.

### TA systems

TA systems in the decaying wood were more represented than in soil, suggesting that wood decomposition may require the action of a higher diversity of microbes. Several functions have been attributed to the TA systems identified in the decaying log and soil and include: PCD (as in the case of mazEF) (Hazan et al., 2001), persistency and growth control (as with phd/doc) (Magnuson, 2007). PCD in the late stages of wood decomposition and in soil can be the result of the competition for specific nutrients and space and implies a threshold density of related microbes present in the microhabitat. PCD may be induced by antagonistic interactions with competitors.

## Conclusions

To our knowledge, this is the first study that has identified genes/molecules, subsystems and microbial taxa which could be considered part of the sociomicrobiome of decaying wood and soils using a metagenomics approach. Other genes, subsystems and microbes can certainly be included in the complex battery of studies regarding the sociomicrobiome. The comparative metagenomic approach in the present study provides ample information on the complex network and interactions of microbes in the considered settings. We present valuable information regarding the microbial ecology of both wood decomposition at its late stages, and that of soil. Results open the opportunity to focus on the expression of specific genes at late stages of wood decomposition, and characterize the molecules associated with the sociomicrobiome of decaying wood and soil. The approach considered here could be applied to study the sociomicrobiome of wood decomposition at earlier stages as well, providing a wider view of the succession of microbes involved in the process.

Given the complexity of the microbial interactions in the ecosystems described here, and the diversity of microbes present, a pertinent question then arises of how microbes detect self (genetically related individuals) from non-self, and how potential mutualistic partners are distinguished from potential competitors. How microbes determine genetically unrelated friend from foe by using non-universal signaling molecules is not clear. Although the presented results are part of the multi microbial interactions that may be involved in wood decay and soil, the battery of genes/molecules identified can be applied to study the sociomicrobiome in other ecosystems.
